# The Leech Condo: An Innovative Approach to Containing Leeches on a Congested Flap

**Published:** 2015-04-08

**Authors:** Stephen L. Viviano, Edward Hahn, Edward S. Lee, Jonathan D. Keith

**Affiliations:** Division of Plastic Surgery, Rutgers—New Jersey Medical School, Newark, NJ

**Keywords:** leech, leech therapy, *Hirudo medicinalis*, venous congestion, free flap salvage

## DESCRIPTION

The patient is a 42-year-old man with a history of glioblastoma resection, external beam radiation, chemotherapy, and cranioplasty. He then developed a chronically draining wound and soft tissue defect that required an anterolateral thigh free flap, which developed venous congestion on postoperative day 1. After surgical exploration, medicinal leech therapy was employed using an innovate approach to contain the leeches in the area of interest.

## QUESTIONS

1. **What are the benefits and challenges of using leeches for a congested free flap?**
2. **What is the authors' innovative method for containing leeches on a flap?**
3. **What other methods for applying and containing leeches have been described in the literature?**
4. **What are the advantages of the “leech condo” over previously described methods?**

## DISCUSSION

**Figure 1:**
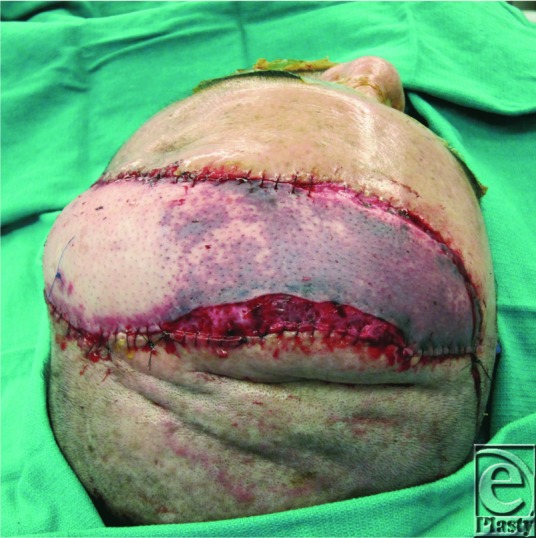
Anterolateral thigh flap forehead reconstruction with area venous congestion before application of “leech condo.”

Leeches are recognized as an effective therapy for venous congestion. Via the anticoagulant hirudin and the removal of excess venous blood, medicinal leeches help prevent the hypoxia, acidosis, and ultimate flap loss that can occur in venous congested flaps. The medicinal leech, *Hirudo medicinalis*, has been used therapeutically for centuries, with the first recorded use dating back to ancient Egypt. In 1955, F. Markwardt isolated the powerful anticoagulant hirudin from leech pharyngeal glands. This discovery renewed modern interest in medicinal leeches,^1^ and by 1960, plastic surgeons began using leeches to treat venous congestion in flaps.^2^ (Fig 1) However, leech therapy presents certain challenges. The leeches must be applied and contained at the site of congestion. Once feeding is complete, the leech then must be removed and a new leech must be applied. This cycle repeats after each leech completes its feed, usually every 60 to 90 minutes. Leeches also have a tendency to migrate, especially after feeding, which can be particularly unsettling to the patient and problematic for the nursing staff. Leech therapy thereby creates a significant logistical burden for the treatment team.

**Figure 2:**
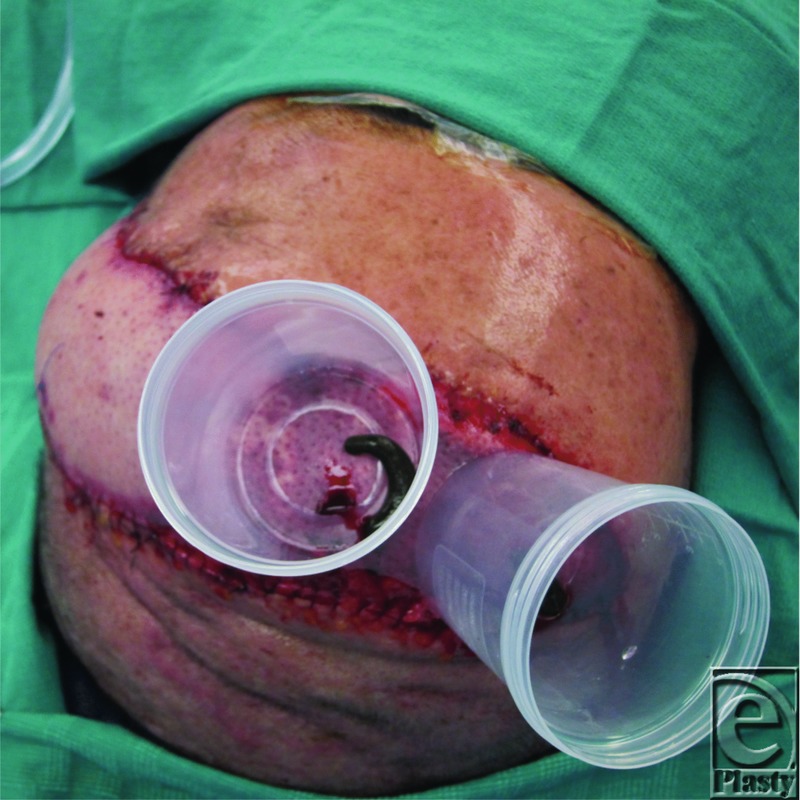
Leech placed into cup and adhered to skin with skin glue.

**Figure 3:**
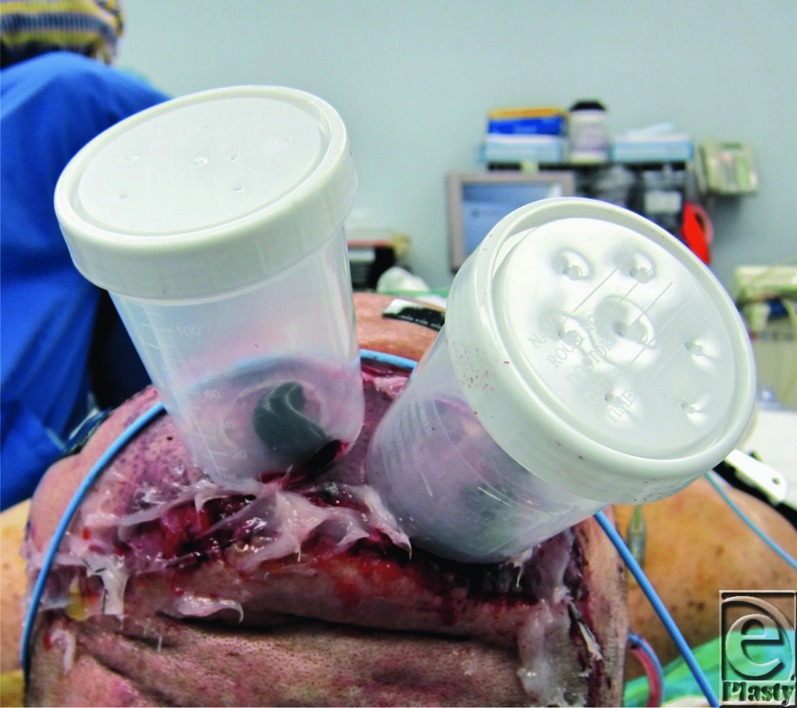
Completed “leech condos” placed over area of venous congestion.

In an effort to minimize these challenges, the authors devised an innovative apparatus, using a plastic screw cap specimen cup to house the leech. First, a circular hole is cut from the bottom of the cup large enough for the leech to attach to the skin. Next, the cup is secured to the area of venous congestion using Dermabond Advanced (Ethicon Inc, Somerville, NJ). Then small holes are punched through the screw cap to allow for airflow, and a small caliber needle is used to produce a drop of blood to encourage the leech to feed. Finally, a leech is placed in the cup (Fig 2), and the cap with air holes is screwed into place (Fig 3).

Other approaches have been reported in the literature. The “leech leash,” as described by Granzow et al^3^ and also practiced by Davila et al,^4^ uses a suture tied through the leech, which is then secured to either the patient's skin or the dressing. Tan et al^5^ construct a “leech cage,” using a small plastic cup with a hole cut from the bottom. The cup is taped to the skin, a leech is inserted, and it is covered with clear plastic film. Azari and Fisher^6^ prefer to use a 3-cc syringe. The plunger is removed, the leech is inserted, and the open end is placed against the skin until the leech attaches. Jagannathan et al^7^ use a similar method but instead secure the syringe to the skin until the leech has finished feeding.

While the previously described methods are reproducible, reliable, and cost-effective, the authors' method provides certain advantages. The specimen cup creates a closed, sealed, and secure trap for the leech. This prevents migration and contains the leech's secretions and excrement, potentially reducing the risk of wound contamination and infection. In addition, the screw cap allows for easy removal of a postprandial leech and reapplication of a new leech without sacrificing the entire apparatus. Once the cup is secured to the patient, it can house multiple cycles of leeches and remain in place for the duration of the therapy. The method also allows for more targeted leech therapy. If only a portion of the flap is congested, as in Figure 1, the leech condo allows for the leech to be delivered directly over the involved portion. While the size of the cup may be a limitation of this technique, the system is versatile and can be deployed to most anatomic regions and to most flap types. In addition, there is little to no morbidity to the application site from the use of the skin glue. The apparatus ultimately allows for more expedient leech management, which decreases the burden on the physicians and nurses.

**Figure 4:**
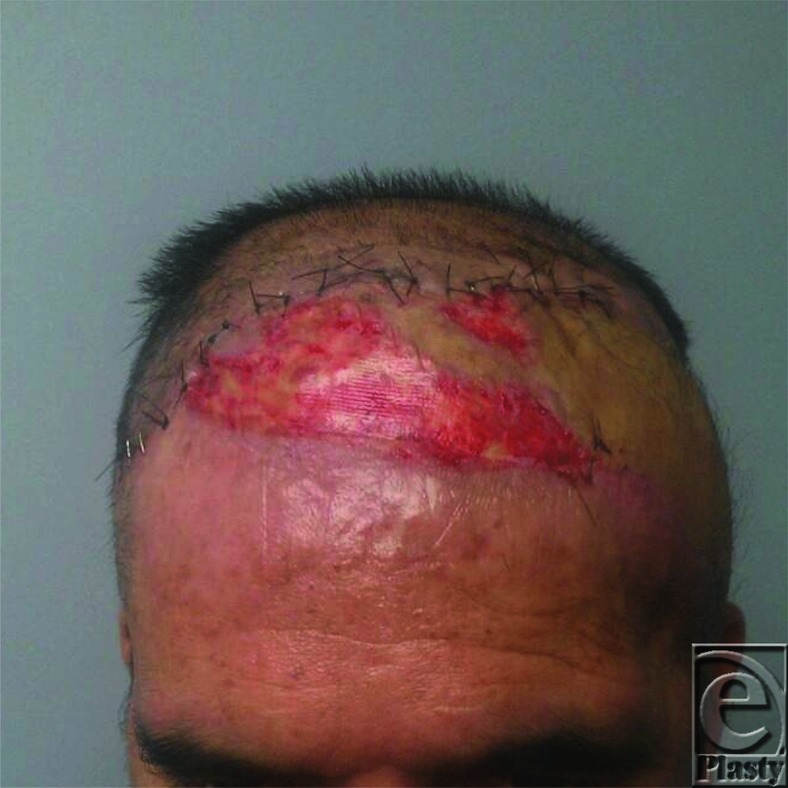
Salvaged, viable flap with minimal epidermal sloughing.

In conclusion, medicinal leech therapy is an effective method for treating venous congestion in free flaps (Fig 4). However, leech therapy is logistically challenging at each step and thus burdens the patient and the health care team. The authors' described method for housing leeches in a modified screw cap specimen cup minimizes the difficulties of leech therapy. Moreover, it provides a more simple, secure, targeted, and expedient system for delivering leeches than most other previously described methods.
